# A 5-Year Follow-Up after Endovascular Treatment of 402 Intracranial Aneurysms—A Single-Centre Experience

**DOI:** 10.3390/biomedicines12061231

**Published:** 2024-06-01

**Authors:** Ana Repić Buličić, David Ozretić, Marko Radoš, Josip Ljevak, Antonela Bazina Martinović, Zdravka Poljaković Skurić

**Affiliations:** 1 Department of Neurology, University Hospital Split, 21000 Split, Croatia; arbulicic@gmail.com; 2 Department of Radiology, University Hospital Zagreb, University of Zagreb School of Medicine, Kišpatićeva 12, 10000 Zagreb, Croatia; 3 Department of Neurology, University Hospital Zagreb, University of Zagreb School of Medicine, Kišpatićeva 12, 10000 Zagreb, Croatia

**Keywords:** intracranial aneurysm, rupture risk, endovascular treatment, complications, retreatment

## Abstract

The aim of our study was to evaluate the early and long-term clinical and morphological outcomes of the endovascular treatment of ruptured and non-ruptured intracranial aneurysms in a cohort of patients from a single centre. We retrospectively analysed the treatment outcomes of 402 endovascularly treated intracranial aneurysms with an average follow-up of 5.5 years. All included patients were treated with endovascular techniques (coil, stent or both). We analysed patient demographics, risk factors for an aneurysm rupture, aneurysm characteristics, and clinical and angiographic complications and outcomes. We analysed and compared the data from the two groups, ruptured aneurysms (RAs) and unruptured aneurysms (UAs), separately. Out of the 318 patients included, a good early clinical outcome was achieved in 78.5% of RAs and in 95.3% of UAs. No complications occurred in 87.71% of patients with UAs and in 80.45% with RAs. The periprocedural rupture rate for UAs and RAs was 0.8% and 2.2%, respectively. The rate of thromboembolic events was 4.8 and 8% for UAs and RAs, respectively. A retreatment due to the recanalisation was required in 9.21% of patients with UAs and in 16.66% of patients with RAs. The results from our centre showed an overall favourable clinical outcome with acceptable periprocedural complications for both RAs and UR aneurysms and proved the endovascular method as safe and effective in the treatment of intracranial aneurysms.

## 1. Introduction

The endovascular treatment of intracranial aneurysms is in many cases a treatment of first choice, preferred even in centres with a neurosurgical treatment option. The main goal of an aneurysm treatment is to prevent its rupture (or re-rupture) by excluding it from the circulation. The procedure can be performed using either endovascular or neurosurgical techniques. The choice of treatment depends mainly on the characteristics of the aneurysm and the clinical condition of the patient, but also on the experience and skills of the individual centre [1,2,3,4].

Even under ideal conditions, a uniform approach to the treatment of unruptured asymptomatic intracranial aneurysms (UAIAs) has not yet been achieved, and their natural history is not fully understood [5,6,7,8]. As UAIAs are relatively common in the general population (with a presumed overall incidence of 0.2–10%), any contribution to predicting their further development could help in deciding on the modality of therapy [9,10,11,12,13]. On the other hand, the International Subarachnoid Aneurysm Trial (ISAT), the largest multicentre randomised controlled trial to date, aimed to evaluate the safety and efficacy of the endovascular coiling treatment compared with neurosurgical clipping in patients with a ruptured aneurysm. The study, which aimed to assess the safety and efficacy of endovascular coiling compared with neurosurgical clipping in patients with ruptured aneurysms suitable for both treatments, showed that endovascular treatment resulted in significantly lower mortality and morbidity than the neurosurgical clipping at a one-year follow-up [1,14].

In this article, we present the results of a mean follow-up of 5.5 years (66 months) for 402 intracranial aneurysms treated with endovascular techniques at our centre and assess the risk factors associated with the aneurysm rupture and the outcome of the endovascular treatment.

## 2. Materials and Methods

In this retrospective study, we present the results of 318 patients with a total of 402 intracranial aneurysms diagnosed and treated at the ESO-certified Comprehensive Stroke Centre of the University Hospital Zagreb between January 2011 and July 2016. We included all the patients diagnosed with intracranial aneurysms, both unruptured and ruptured, treated with endovascular techniques at our centre. Patients treated by neurosurgical clipping (156 aneurysms in 156 patients) and patients lost to follow-up were excluded. We also excluded patients with infectious aneurysms and aneurysms in combination with arteriovenous malformations, fistulas or moyamoya disease as well as patients with incomplete documentation (e.g., missing DSA in cases where patients died before the procedure).

We analysed the aneurysm’s characteristics (its location, size, rupture status, including morphological changes during the follow-up period), patient characteristics (demographics and two risk factors: smoking and hypertension) and treatment outcomes (morbidity, mortality and a need for follow-up). We analysed and compared the data from two groups, ruptured and unruptured aneurysms, separately. In addition, the occurrence and the type (ischemic, haemorrhagic or migration of endovascular material) of periprocedural complications and late complications were recorded.

The primary outcomes of the study were the neat result after the endovascular embolisation of the aneurysm and the mortality and morbidity rates in patients treated with the endovascular treatment method. The secondary outcomes were periprocedural complications and retreatment rate. A successful endovascular treatment was defined as a complete occlusion of the aneurysm. A functional clinical outcome was defined by the modified Rankin Score (mRS) [15]. In the literature, the mRS is considered a practical and relevant tool for assessing functional outcomes after cerebral vascular incidents [16]. It consists of seven well-defined degrees of disability (from 0 for no disability to 6 for death). It is the most commonly used scale in randomised control trials for patients with aneurysmal SAH [17]. The morbidity rate was defined as mRS grade 3–5, and the mortality rate was defined as mRS grade 6.

The patients were followed up for at least 3 and up to 82 months. The regular follow-up protocol consisted of a clinical and angiographic examination. A control magnetic resonance angiography (MRA) was performed three months after the treatment, followed by a digital subtraction angiography (DSA), which was performed after a period of 6–12 months, depending on the findings of the first MRA check. Both untreated and treated aneurysms with stable results were subsequently examined annually using one of the non-invasive angiography methods, usually MRA. In the case of growth or change in the morphology, DSA was performed, followed by endovascular treatment of the traced aneurysm. The clinical status of the patients was assessed using the modified Rankin scale during regular visits by a stroke neurologist.

The datasets were analysed depending on the measurement scale used. Univariate analysis was performed using the chi-square test (χ^2^) and Fisher’s exact test (p) for categorical variables. Assumptions for these tests include categorical data, mutually exclusive categories and independence of the study groups. In addition, Student’s *t*-test with acceptable values for skewness and kurtosis was calculated for continuous data. For ordinal datasets, the non-parametric Mann–Whitney U test, expressed as a Z-score, was presented. Spearman’s rank-order correlation (Spearman’s ρ) was used to compare the relationship between ordinal or rank-ordered variables. Point-biseral correlations (rpb) were performed to compare the relationship between two variables when one of the variables was dichotomous. Binary logistic regression analysis assuming an association between a set of independent variables and a binary dependent variable was used to test the predictors of re-embolisation (poor outcome-mRS ≥ 4, complications and retreatment). Results were expressed as odds ratios (ORs) with 95% confidence intervals (CIs) and *p* values. Descriptive statistics were summarised as N, percentage, mean and standard deviations. A threshold of *p* < 0.05 was used to determine the significance level of the effect. Data analysis was performed using Statistica 12 software.

## 3. Results

### 3.1. Participants

Of the 318 patients with an aneurysm who participated in this study, 73.58% were women and 26.42% were men. The mean age of the participants was 54.13 ± 12.31 years (Mfemale = 55.61 ± 12.08; Mmale = 50.56 ± 10.81) with an age range from 18 to 89 years. Overall, 56.71% of patients had unruptured intracranial aneurysms, and 43.28% had ruptured aneurysms. The majority of intracranial aneurysms reported in patients were solitary (57.96%), and 42.03% were multiple.

### 3.2. Results

In total, 228 UAs and 174 RAs were reported in patients. No significant difference was found in the mean age between the patients with UAs and RAs (t = 0.31; *p* = 0.75). In the gender ratio, significantly more female participants (χ^2^ = 12.7; *p* < 0.001) were found in the group of RAs (UA = 50.78%; RA= 68.60%). The difference between the groups of UAs and RAs concerning hypertension (χ^2^ = 0.58; *p* = 0.44) and smoking habits (χ^2^ = 2.23; *p* = 0.32) was not significant.

Table 1 compares the characteristics of 228 recorded UAs and 174 RAs. Significant differences between the two groups were found in the aneurysm’s type (solitary/multiple) (χ^2^ = 16.88; *p* < 0.001) and aneurysm’s location (Z = −6.12; *p* < 0.001). Multiple aneurysms were more represented in UAs (50.88%) than in RAs (30.46%). Regarding the location, most of the aneurysms in UAs are located on ACI (63.59%) and PO (10.96%). In RAs, the aneurysm location was mostly on ACoA (26.43%) and ACI (25.86%). The size range differences of aneurysms between the two groups were not significant (Z = −1.38; *p* = 0.16) (Table 1). The endovascular embolisation outcome between the groups of UAs and RAs was not significant (χ^2^ = 1.15; *p* = 0.28). The majority of patients have good outcome results (UA = 66.67%; RA = 61.49%). For the aneurysm outcome, a significantly higher retreatment rate as a parameter of successful embolisation treatment (χ^2^ = 5.04; *p* = 0.02) was found in the group of RAs (UA = 9.21%; RA = 16.66%).

**Figure 1:**
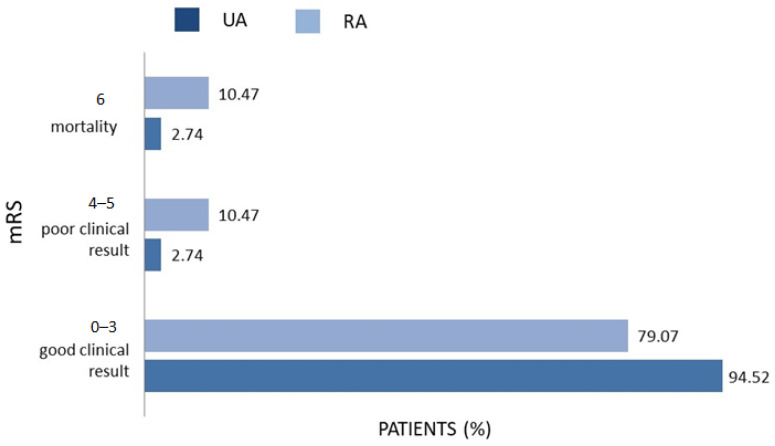
Distribution of mRS scores on discharge in UA (N = 228) and RA group (N = 174). mRS—modified Rankin Scale (0–6).

**Figure 2:**
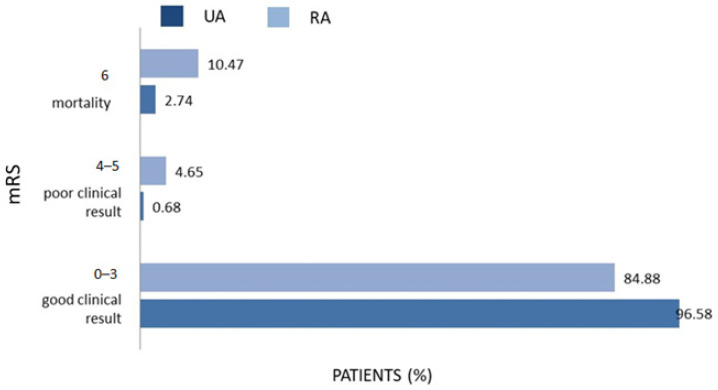
Distribution of mRS scores at the end of patient follow-up in UA (N = 228) and RA group (N = 174). mRS—modified Rankin Scale (0–6).

The patients with RAs had significantly higher mRS scores on discharge (10.47%) and at the end of the patient’s follow-up (10.47%) than the UA group (2.74%). In Figure 1 and Figure 2, the distribution of mRS scores on discharge and at the end of the follow-up is presented.

In Table 2, correlations between the relevant clinical variables for all patients are presented (N = 318). A significant but weak correlation was found between gender and the rupture of an aneurysm (r = 0.12; *p* < 0.05). Furthermore, the rupture of an aneurysm is correlated significantly with the location of the aneurysm (r = 0.30; *p* < 0.05), mRS on discharge (r = 0.21; *p* < 0.05) and mRS at the end of the patient’s follow-up (r = 0.18; *p* < 0.05). The results indicate no significant correlation between the re-embolisation (r = 0.11; *p* > 0.05) and periprocedural complications with the rupture of an aneurysm (r = 0.09; *p* = 0.08; *p* > 0.05).

### 3.3. Predictors of Clinical Outcome

In the binary logistic regression analysis, the rupture of an aneurysm (OR = 2.20; *p* = 0.04; CI = 1.01–4.82), gender (OR = 2.41; *p* = 0.03; CI = 1.06–5.44) and the type of aneurysm (solitaire/multiple) (OR = 3.47; *p* = 0.001; CI = 1.63–7.39) remained a significant predictor of re-embolisation (embolisation treatment success parameter) (Table 3). The rupture of an aneurysm was a significant predictor of a poor clinical outcome (OR = 0.02; *p* < 0.001; CI = 0.01–0.10). Furthermore, the diameter was found to be a significant predictor of a poor outcome (OR = 1.82; *p* < 0.001; CI = 1.43–2.31), complications (OR = 1.33; *p* = 0.05; CI = 1.09–1.63) and retreatment (OR = 1.49; *p* < 0.001; CI = 1.19–1.86) (Table 3).

## 4. Discussion

Demographic data from previous studies have shown that aneurysms mostly occur in the population aged 50–55 years, with a slight preponderance of women, while 20–30% of patients had multiple aneurysms [11,18,19,20,21].

Our patient cohort showed similar characteristics in terms of the age of the patients and the predominance of women. However, we found a slightly higher percentage of multiple aneurysms (42.03%) in our patient cohort, which could be a result of the population characteristics or more detailed neuroimaging.

The risk factors for an aneurysm formation and rupture have previously been reported to be age, female sex, smoking and hypertension [22]. In the present study, there were also more patients with hypertension and smoking habits in the patients with RAs than in the UA group, but statistical analysis showed no statistical significance for these risk factors between the groups. Although some studies reported that age [7] and multiple aneurysms [23] were the risk factors for an aneurysm rupture, our study did not show the same result.

In a large prospective study of unruptured aneurysms, the International Study of Unruptured Intracranial Aneurysms (ISIUA), posterior circulation aneurysms were reported to have a higher risk of rupture [24].

In our study, aneurysms in the anterior circulation, especially ACoA and ACI, were more likely to rupture. Our finding is more consistent with the UCAS study in the Japanese population by Morita et al. who reported that aneurysms in the anterior and posterior communicating artery had a higher risk of rupture [25].

Two large prospective studies found that the aneurysms with a diameter of less than 10 mm [24] and 7 mm [25] had a low risk of rupture. In contrast, our study showed no significant differences in size between the ruptured and unruptured aneurysms. Also, more than half (57.04%) of the ruptured aneurysms in our cohort were smaller than 7 mm in diameter.

The discrepancy between the sizes of ruptured aneurysms in prospective and retrospective studies is probably due to other factors that influence the risk of a rupture, such as morphology, shape and location. In any case, it has already become clear that size should not be the only decisive factor when choosing the treatment option for an unruptured aneurysm, as it is not decisive for determining the risk of rupture.

### 4.1. Treatment Outcomes

The treatment results of our study are largely consistent with previously published randomised trials [3]. The one-year mortality rate for ruptured aneurysms treated with an endovascular approach was 9% in this review, slightly lower than in our patient group, where we observed a mortality rate of 10.4%.

A meta-analysis of 22 studies comparing the neurosurgical clipping and the endovascular coiling of ruptured aneurysms found that neurosurgical clipping provides better outcomes in terms of mortality, rebleeding and retreatment needs, while endovascular coiling has fewer postoperative complications [1].

In a study by Henkes at a single centre, on a total of 1811 aneurysms, both RAs and UAs, 72.2% of UAs and 64.5% of RAs were without neurologic symptoms. Mild and severe permanent deficits occurred in 2.5 and 2.7% of UAs, respectively. No procedure-related complications occurred in 81.1% of UA patients and 83.7% of RA patients [2].

Looking at the early treatment outcomes in our study, a good early clinical outcome was achieved in 95.3% of patients with UAs and 78.5% of patients with RAs. As the clinical outcome of an RA is strongly influenced by the complications of the disease itself, postprocedural treatment protocols in our centre should be encountered as well. Our approach to vasospasm treatment is rather conservative (neuroprotective agents and avoiding blood pressure instability by maintaining MAP between 80 and 100 mmHg) than interventional (which is used only if proven vasospasm of a large intracranial vessel occurs). Although different, there is still no proof of the best treatment approach [26]. Finally, outcomes remained stable or even improved in the majority of patients during an average follow-up period of 5.5 years. Thus, the proportion of patients with poor outcomes in UA at the end of the follow-up was 2.7%, which is in the range of the previously reported 2–4% [4].

### 4.2. Periprocedural Complications

Despite improvements in endovascular devices and an increasing clinical experience, the endovascular treatment of intracranial aneurysms still carries a risk of neurological complications. The most common complications are ischemic events and an intraprocedural rupture of the aneurysm [27,28,29].

Regarding the ischemic events during RA treatment, our study shows a lower rate (8.04%) than the 12.5% previously reported by Pierot et al. [30]. An intraprocedural rupture rate occurred in 2.29% of patients in our study for RAs, in contrast to 5% in the CARAT study by Elijovich et al. [31] and similar to the 2,5% reported by Sluzewski et al. [32].

Many other authors also reported periprocedural complications of less than 10% overall and a good clinical outcome in 80% or more [33,34,35,36]. Brilstra et al. reported a complication rate of 12%, aneurysm rupture of 2.4% and ischemic events of 8.5% in a series of 1256 aneurysms treated with RA [3].

Jiang et al. report the results of neurosurgical and endovascular treatment of UAs and RAs in a meta-analysis [37]. The results of this meta-analysis are somewhat contradictory. Surgical clipping may be superior to endovascular coiling in RAs, but clipping on the other hand was associated with a greater incidence of poor outcomes and bleeding compared to coiling in UAs. Retreatment was performed in 56 cases (3.5%) after clipping and 258 times (16.0%) after initial coiling, which is a higher rate than reported in the present study. However, in this meta-analysis, aneurysm characteristics such as size and location were not analysed, which may have an effect on the choice of treatment and periprocedural complications and subsequently change the results. Another possible explanation is that the results of RAs in our study may be confounded by the loss of patients to follow-up.

If we compare our results with those already published, we can conclude that the endovascular approach is a safe and effective method for ruptured aneurysms.

For unruptured aneurysms, the situation is more controversial as there are no randomised controlled trials. Reports from the observational studies have mostly found lower morbidity and mortality rates than with surgical clipping [35,37,38,39].

In a meta-analysis of 114 studies on endovascular and neurosurgical treatments of UAs, periprocedural complications leading to temporary or permanent clinical deterioration were 4.96% for the endovascular and 8.34% for the neurosurgical treatment. Factors associated with complications in the endovascular treatment were female gender, diabetes, hyperlipidemia, wide neck, posterior circulation, stent-assisted coiling and stenting [40].

The rate of ischemic events in UA in our study was 4.82%, and the aneurysm rupture rate was 0.87%, which is also lower than in previous reports [41,42,43].

Two meta-analyses with long-term follow-up (>3 years), one by Krag et al. for both RAs and UAs and one by Huselberg et al. for UAs, reported better long-term durability of clipping compared to coiling [44,45]. This is in contrast to the present study, which shows a lower rate of retreatment compared to the data reported in these meta-analyses. One possible explanation for this discrepancy is that both meta-analyses only examined coiling EVT, while the modern EVT techniques were not analysed.

Kang et al. reported a lower recurrence rate in UAs treated with neurosurgical clipping compared with endovascular coiling (endovascular coiling 19.0% vs. surgical clipping 8.3%) and a lower incidence of periprocedural complications (coiling vs. clipping 4.60% vs. 7.0%) [46]. However, the follow-up time in this meta-analysis was 1 year, and no patient characteristics were analysed. Both could lead to different results compared to our study. We found that the aneurysm size (diameter) was the risk factor associated with periprocedural complications in our cohort. Pierot et al. [43] found similar results: larger aneurysms are associated with an increased risk of a periprocedural ischemic stroke. One possible explanation is that larger aneurysms are more likely to contain clots that could be displaced into the distal branches during the procedure and cause an embolism [42].

### 4.3. Retreatment

The main reason for a long-term follow-up after the endovascular treatment of an aneurysm is the possibility of its recanalisation and the risk of the appearance or growth of a new aneurysm.

Recurrence rates after EVT reported in previous studies range from 10 to 30% [47,48,49,50,51,52,53,54]. In our cohort, additional endovascular intervention was required in 12.4% of aneurysms, which was on the lower end of this range. Also, ruptured aneurysms had a higher retreatment rate compared to non-ruptured aneurysms (9.21 vs. 16.6%). This was consistent with the results of previous reports [47,49,55]. Our patient cohort showed a higher retreatment rate in female patients, in patients with multiple aneurysms and in patients with larger aneurysms.

According to a previously published meta-analysis by Froelich et al. [55], a higher retreatment rate was found for aneurysms in the vertebrobasilar arteries and PCA. The systematic review by Ferns et al. [47] also reported that the aneurysms in the posterior circulation were retreated more frequently. It is possible that aneurysms of the posterior circulation with complicated anatomy were more frequently treated with endovascular and less with neurosurgical methods [47]. In our study, no significant correlation was found between the number of treatments and the location of the aneurysms. The peculiarity of the present study was that most of the aneurysms were located in the anterior circulation, which could be one of the reasons for this.

Aneurysm growth is one of the proposed mechanisms for aneurysm recanalisation after successful endovascular treatment [56,57,58]. During the follow-up, aneurysm growth was detected in only 1.75% of the UA group and not in the RA group. A low rate of aneurysm growth suggests that other mechanisms such as coil compaction may have played an important role. A more detailed analysis of the aneurysm morphology is needed to examine this issue.

For the subgroup analysis, we found statistically significant differences in periprocedural complications and retreatment rates between the RA and UA groups. We can speculate that more thromboembolic events in the RA group, as the main complication, could be due to changes in the coagulation system, as reported by Jun et al. [59]. Also, rebleeding from the ruptured aneurysms during the procedure is also more common in RAs [31]. On the other side, stents and stent-assisted coiling, also connected with more thromboembolic events, were used mostly in the UA group, but adequate antiplatelet therapy before treatment reduces the rate of these complications. Similar results are found in the study by Ihn et al. [42]. These results can implicate different treatment modalities for RAs and UAs.

## 5. Conclusions

The results of the treatment of aneurysms by endovascular embolisation in our study showed an overall favourable clinical outcome with few periprocedural complications in both RAs and UR aneurysms.

Although size did not correlate with a rupture risk, larger diameter aneurysms had a worse clinical outcome, had more complications during the procedure and were retreated more frequently. The study by Yang et al. also found a worse clinical outcome in older people with larger aneurysms [60]. In accordance with these results, we can assume that endovascular treatment may not be preferable for large and giant aneurysms. On the other hand, the meta-analysis by Dengler et al. shows similar results for both neurosurgical and endovascular types of treatment for giant aneurysms [61]. The study by Santoro et al. showed similar results but a higher percentage of periprocedural complications in the neurosurgical group compared to the endovascular treatment group [62], so further research on the relationship between aneurysm size and treatment choice is needed to draw more accurate conclusions.

In addition, our study confirmed that several parameters should be considered when assessing the risk of a rupture or endovascular treatment, while the size of the aneurysm probably does not play a decisive role.

A limitation of this study is that it is a single-centre retrospective study with the possibility of selection bias. The fact that our centre is a tertiary treatment centre has resulted in some patients being referred to smaller centres after the initial treatment and being lost to follow-up. Another limitation is that we did not analyse the morphological features of the aneurysms. The shape of the aneurysm may be a confounding factor in the interpretation of the results of our study. In addition, the present study did not differentiate between the different coiling techniques. Future studies based on the analysis of different endovascular methods are needed to draw more accurate conclusions about the long-term results of this treatment.

More structural prospective studies that include well-defined aneurysm morphology (shape) together with patient characteristics will be needed in the future. These results could lead to a better selection of patients who will benefit most from the endovascular therapy.

On the other hand, there are still knowledge gaps regarding modern endovascular techniques that still need to be evaluated by long-term studies to explore their durability and the need for additional treatments and also to compare them with neurosurgical treatments.

We believe that the results of our study, despite its limitations, can be useful in everyday clinical practice. Current practice guidelines suggest surgical treatment for younger patients to avoid possible risks of retreatment during their lifetime and endovascular treatment for older patients with comorbidities. A future investigation of the durability and safety of the endovascular treatment could implicate changes in future management strategies.

The data generated by the present study could be useful in discussions with patients and their families about the risks and benefits of endovascular treatment at our centre.

## Figures and Tables

**Table 1:** Clinical features of unruptured and ruptured intracranial aneurysms.

	Unruptured (N = 228)	Ruptured (N = 174)	Test	*p* Value
Aneurysm				
Solitary, n (%)	112 (49.12)	121 (69.54)	χ^2^ = 16.88	<0.001 **
Location, n (%)				
ACI	145 (63.59)	45 (25.86)	Z = −6.12	<0.001 **
ACA	12 (5.26)	11 (6.32)		
ACoA	17 (7.45)	46 (26.43)		
ACM	9 (3.94)	4 (2.29)		
BA	10 (4.38)	21 (12.06)		
ACoP	10 (4.38)	24 (13.79)		
PO	25 (10.96)	23 (13.21)		
Size range (mm), n (%)				
<3	28 (12.90)	7 (4.29)	Z = −1.38	0.16
3–4.99	64 (29.49)	29 (17.79)		
5–6.99	32 (14.74)	57 (34.96)		
7–9.99	38 (17.51)	43 (26.38)		
10–14.99	29 (13.36)	18 (11.04)		
>15	26 (11.98)	9 (5.52)		
Embolisation outcome, n (%)				
Good results	152 (66.67)	107 (61.49)	χ^2^ = 1.15	0.28
Retreatments, n (%)	21 (9.21)	29 (16.66)	χ^2^ = 5.04	0.02 *
Recanalisation up to 3 mm, n (%)	56 (24.56)	36 (20.68)	χ^2^ = 0.84	0.35
Aneurysm progression, n (%)	4 (1.75)	0 (0)	χ^2^ = 3.08	0.07
Postprocedural events, n (%)				
None	200 (87.71)	140 (80.45)	Z = −2.01	0.04 *
Stent migrations	8 (3.50)	4 (2.29)		
Ischemia	11 (4.82)	14 (8.04)		
Rupture	2 (0.87)	4 (2.29)		
Postprocedural hydrocephalus	2 (0.87)	0 (0)		
Treatment, n (%)				
Solo coil	135 (77.58)	154 (88.50)	Z = 6.57	<0.001 **
Coil + SAH	68 (29.82)	18 (10.34)		
Solo stent	25 (10.96)	2 (1.14)		

Abbreviations: ACI—internal carotid artery; ACA—anterior cerebral artery; ACoA—anterior communicating artery, ACM—middle cerebral artery, BA—basilar artery; ACoP—posterior communicating artery; PO—other arteries of posterior circulation; SAH—*Subarachnoid hemorrhage*; 4.8% of missing data in UIA and 6.3% of missing data in RIA; * *p* < 0.05; ** *p* < 0.001.

**Table 2:** Correlations (ρ, rpb) between relevant clinical variables (N = 318).

	UA/RA
Gender	0.12 *
Smoking	−0.05
Hypertension	−0.00
Solitary/Multiple	0.07
Location	0.30 *
Size	−0.11
Late complications	−0.02
Retreatments	0.11
mRS discharge	0.21 *
mRS end of the patient follow-up	0.18 *
Periprocedural complications	0.10 *

Abbreviations: * *p* < 0.05.

**Table 3:** Univariate binary logistic regression analyses with a poor outcome (mRs ≥ 4), complications and retreatments as a dependent variable.

	mRs ≥ 4			Complications			Retreatment		
Predictor	OR	*p* Value	95% CI	OR	*p* Value	95% CI	OR	*p* Value	95% CI
Rupture	0.02	<0.001 **	0.01–0.10	0.59	0.06	0.34–1.02	2.20	0.04 *	1.01–4.82
Gender	0.57	0.09	0.29–1.11	0.68	0.20	0.37–1.23	2.41	0.03 *	1.06–5.44
Age	0.99	0.73	0.97–1.02	1.01	0.93	0.97–1.02	1.0	0.53	0.97–1.04
Solitary/Multiple	-	0.99	-	1.22	0.48	0.69–2.15	3.47	<0.001 **	1.63–7.39
Smoking	0.79	0.29	0.51–1.22	0.80	0.35	0.49–1.28	1.2	0.59	0.63–2.23
Hypertension	1.22	0.45	0.73–2.04	1.56	0.11	0.89–2.76	0.86	0.72	0.38–1.95
Location	1.09	0.15	0.96–1.23	1.07	0.26	0.95–1.20	0.87	0.18	0.72–1.06
Diameter	1.82	<0.001 **	1.43–2.31	1.33	0.05 *	1.09–1.63	1.49	<0.001 **	1.19–1.86

Abbreviations: * *p* < 0.05; ** *p* < 0.001.

## Data Availability

The data are available within the article.
